# A decade-long real-world cohort (2016–2025): development of an individualized risk-stratification nomogram and evaluation of clinical utility for recurrent respiratory tract infections in children

**DOI:** 10.3389/fped.2026.1806366

**Published:** 2026-05-12

**Authors:** Hehua Cai, Juan Li, Hui Wang

**Affiliations:** Department of Pediatrics, The Third People’s Hospital of Chengdu, Chengdu, Sichuan, China

**Keywords:** children, clinical utility, logistic regression, nomogram, prediction model, recurrent respiratory tract infections, risk stratification

## Abstract

**Background:**

Recurrent respiratory tract infections (RRTIs) are common in children and are associated with substantial healthcare costs and reduced quality of life. A practical tool for individualized risk assessment may facilitate the early identification of high-risk children and support targeted prevention.

**Methods:**

In this 10-year real-world cohort study (2016–2025), eligible children were screened and followed according to predefined criteria. After exclusions, 6,026 children were included, of whom 1,227 (20.4%) developed RRTIs. The cohort was randomly divided into a training set (*n* = 4,219; events = 859) and a testing set (*n* = 1,807; events = 368). Multivariable logistic regression was used in the training set to identify independent predictors and develop a customized nomogram. Bootstrap resampling was used for internal validation, and model performance was assessed in terms of discrimination, calibration, and clinical utility using decision-curve analysis.

**Results:**

Multivariable analysis identified history of allergy (OR 5.187), history of asthma (OR 2.522), lower vitamin A level (OR 0.458 per 0.1 mg/L increase), lower vitamin D level (OR 0.556 per 10 ng/mL increase), lower birth weight (OR 0.283 per kg increase), passive smoking exposure (OR 2.061), and lower hemoglobin level (OR 0.425 per 10 g/L increase) as independent predictors of RRTI (all *P* < 0.001). The nomogram demonstrated good discrimination and calibration in both the training and testing sets. Decision-curve analysis supported its potential clinical utility by showing a favorable net benefit across clinically relevant threshold probabilities. Risk stratification based on predicted-probability cutoffs further separated children into low-, intermediate-, and high-risk groups with clearly different observed event rates.

**Conclusion:**

We developed and validated a nomogram based on routinely available clinical history and nutritional indicators to predict individualized RRTI risk in children. This tool demonstrated robust predictive performance and may support early risk stratification and preventive decision-making in pediatric practice.

## 1 Introduction

Recurrent respiratory tract infections (RRTIs) remain one of the most common reasons for pediatric outpatient visits and hospital admissions (1). Beyond repeated episodes of cough, wheeze, and fever, RRTIs may contribute to poor growth, prolonged school absenteeism, caregiver anxiety, and substantial healthcare utilization (2), particularly when infections are persistent or complicated. In some regions of China, epidemiological surveys have reported that the proportion of children experiencing RRTIs may be as high as approximately 30% (3), highlighting an important and ongoing public health burden.

The development of RRTIs is multifactorial (4). Previous studies and clinical experience suggest that host susceptibility (e.g., allergic predisposition and asthma) (5), early-life characteristics (e.g., low birth weight), environmental exposures (e.g., passive smoking exposure) (6), and nutritional or hematologic status (e.g., deficiencies in fat-soluble vitamins and low hemoglobin levels) (7) may all contribute to an increased risk of recurrent infections. However, in routine clinical practice, these factors often coexist and interact, making it difficult to translate group-level associations into individualized risk estimates for a specific child at the point of care.

Accurate individualized prediction is clinically meaningful because preventive strategies (8), such as parental counseling regarding smoke exposure, nutritional optimization, and closer follow-up for children with atopy or asthma, are more feasible and cost-effective when targeted to those at highest risk. In recent years, clinical prediction models, particularly nomograms, have been increasingly used (9) to provide intuitive, bedside-friendly probability estimates that support shared decision-making and early preventive intervention. Nevertheless, robust real-world models tailored to pediatric RRTIs, with clear evaluation of clinical utility, remain limited (4), and model performance may vary across regions because of differences in population characteristics and healthcare practices.

We therefore aimed to (1) identify independent predictors associated with RRTIs in children, (2) develop an individualized risk-stratification nomogram, and (3) comprehensively evaluate model discrimination, calibration, and clinical utility to facilitate the early identification of high-risk children and support practical prevention strategies in pediatric care. To achieve these aims, we used a decade-long real-world cohort.

## 2 Materials and methods

### 2.1 Study design and setting

This study was a decade-long real-world cohort analysis of children with respiratory tract infections managed in the Department of Pediatrics at our center between January 1, 2016 and January 1, 2025. The study aimed to develop an individualized risk-stratification nomogram for recurrent respiratory tract infections (RRTIs) and to evaluate model performance and clinical utility.

The Third People's Hospital of Chengdu is a national tertiary Grade A general hospital located in Chengdu, Sichuan Province, China. It provides medical care, teaching, research, prevention, and rehabilitation services, and the Department of Pediatrics offers routine outpatient and inpatient care for children from the local urban community as well as nearby areas. Accordingly, this cohort reflects a real-world pediatric population encountered in routine clinical practice at a high-volume comprehensive medical center.

### 2.2 Participants

Eligibility screening was performed for children who presented with or were hospitalized for respiratory tract infection during the study period. The inclusion criteria were age ≤14 years, a diagnosis of respiratory tract infection at presentation, and complete clinical and laboratory data required for model development. The upper age limit of 14 years was selected because the study was conducted in the pediatric department of our institution, where children aged 14 years or younger are routinely managed in clinical practice. In addition, the outcome definition used in this study was based on age-specific RRTI criteria applicable to children aged 0–14 years. For the purpose of cohort entry, respiratory tract infection was defined as an acute infectious disease involving the upper and/or lower respiratory tract at the index visit/admission, identified from the electronic medical record according to clinician-documented diagnoses and corresponding clinical assessment. These diagnoses included upper respiratory tract infection, bronchitis, bronchiolitis, pneumonia, and other acute respiratory infections routinely managed in pediatric practice. Upper respiratory tract infections and lower respiratory tract infections were classified according to the treating clinician's final diagnosis documented in the medical record rather than on isolated symptoms alone. In routine pediatric practice at our center, this classification was based on the overall clinical assessment, including presenting symptoms, physical examination findings, and, when clinically indicated, auxiliary examinations such as chest x-ray. Episodes diagnosed as common cold, rhinitis, pharyngitis, tonsillitis, sinusitis, or otitis media were classified as upper respiratory tract infections, whereas episodes diagnosed as bronchitis, bronchiolitis, or pneumonia were classified as lower respiratory tract infections. In particular, lower respiratory tract infection was generally supported by evidence of lower airway or pulmonary involvement on clinical examination, such as abnormal lung auscultation findings, and/or radiographic findings when available. Chronic non-infectious airway diseases were not considered qualifying infectious diagnoses; therefore, asthma history was treated as a baseline comorbidity/predictor rather than as an index respiratory tract infection diagnosis. The exclusion criteria were congenital pulmonary hypoplasia, severe liver or kidney failure, malignant tumors, and loss to follow-up. In addition, documented congenital immunodeficiency was not available as a complete standardized standalone variable for the full retrospective cohort; however, when severe congenital immunodeficiency or other major underlying diseases predisposing to recurrent infection were clearly documented in the medical record, such children were not intended for inclusion in the analytic cohort. Of the 6,200 children initially assessed, 174 were excluded (lost to follow-up, *n* = 74; congenital pulmonary hypoplasia, *n* = 43; severe hepatic/renal dysfunction, *n* = 27; malignant tumor, *n* = 30), leaving 6,026 children in the final analytic cohort.

### 2.3 Follow-up and outcome definition

All eligible children received standard-of-care management and were followed for 1 year after the index visit/admission through outpatient visits and/or telephone or online contact. The primary outcome was RRTI during follow-up, defined as recurrent upper and/or lower respiratory tract infections exceeding age-specific thresholds (3, 10) within 1 year. In this study, upper respiratory tract infections included recurrent infectious episodes such as the common cold, pharyngitis, tonsillitis, rhinitis, sinusitis, and otitis media, whereas lower respiratory tract infections included bronchitis, bronchiolitis, and pneumonia diagnosed during follow-up. Classification at follow-up was based on the principal diagnosis recorded by the treating clinician for each infectious episode rather than on isolated symptoms such as cough or nasal discharge alone. In routine clinical practice at our center, the principal diagnosis was established using the combination of symptoms, physical examination findings, and, where clinically required, chest imaging results or other relevant auxiliary examinations. Accordingly, when lower respiratory diagnoses such as bronchitis, bronchiolitis, or pneumonia were documented, especially with evidence of lower airway or pulmonary involvement on examination (e.g., abnormal lung auscultation findings) and/or radiographic findings when available, the episode was categorized as a lower respiratory tract infection; episodes diagnosed as common cold, rhinitis, pharyngitis, tonsillitis, sinusitis, or otitis media were categorized as upper respiratory tract infections. RRTI was considered present when the annual episode frequency exceeded the following predefined thresholds: upper respiratory tract infection >7 episodes/year for ages 0–2 years, >6 episodes/year for ages 3–5 years, and >5 episodes/year for ages 6–14 years; lower respiratory tract infection ≥2 episodes/year for ages 0–14 years. A history of asthma or allergy was considered present only when previously diagnosed by specialist physicians (11).

### 2.4 Candidate predictors and data collection

Candidate predictors were prespecified on the basis of prior literature and clinical plausibility (12) and were extracted from the electronic medical record and laboratory information system. Clinical variables included sex, age, body mass index, residence, family history of chronic respiratory disease, delivery mode, perinatal asphyxia, preterm birth status, breastfeeding duration, outdoor activity time, and passive smoking exposure. Residence was classified as an urban or rural living environment according to the recorded residential information in the medical record and was examined as a candidate environmental predictor. Key comorbidities included physician-diagnosed allergy and asthma. Laboratory variables focused on nutritional and hematologic indicators, including vitamin A, vitamin D, hemoglobin, and other routinely measured micronutrient-related indices when available. Continuous variables were recorded as measured values, and categorical variables were coded as binary or nominal categories, as appropriate. Hospitalization-related management/severity variables at the index episode, including chest x-ray findings, oxygen requirement, length of hospitalization, antibiotic treatment, and other detailed in-hospital interventions, were not consistently available in a standardized format across the full cohort and were therefore not included in the present model. Etiologic examinations to distinguish viral from bacterial respiratory tract infections were not systematically performed or recorded in a standardized manner across the full cohort; therefore, viral-vs.-bacterial proportions could not be reliably determined for the present analysis.

### 2.5 Model development and nomogram construction

A training set (70%, *n* = 4,219) and a testing set (30%, *n* = 1,807) were randomly selected from the cohort. Independent predictors of RRTI in the training set were identified using multivariable logistic regression. For multivariable modeling, variables with statistical support in univariable analyses and/or clear clinical relevance were considered, and collinearity was assessed before final model fitting (13). A nomogram was then constructed using the regression coefficients from the final model to estimate the individualized probability of RRTI.

### 2.6 Model performance, validation, and clinical utility

Discrimination was assessed using the concordance index (C-index) and the area under the receiver operating characteristic curve (AUC) (4). Internal validation in the training set was performed using bootstrap resampling (1,000 iterations) (14), and calibration was assessed using calibration plots (15) comparing predicted and observed risks. Hold-out validation was performed in the testing set. Decision-curve analysis (DCA) (16) was used to estimate net benefit across a range of threshold probabilities to evaluate clinical utility. Risk-stratification analyses were further performed by classifying children into risk groups according to predicted probabilities and comparing observed event rates across strata.

### 2.7 Statistical analysis

Statistical analyses were performed using Python. Continuous variables are presented as mean ± standard deviation or median (interquartile range), as appropriate, and were compared using the Student's *t*-test or Mann–Whitney U test. Categorical variables are presented as counts (percentages) and were compared using the chi-square test or Fisher's exact test, as appropriate. All tests were two-sided, and *P* values < 0.05 were considered statistically significant. Model development and validation were conducted in accordance with accepted reporting guidelines for prediction models.

## 3 Results

### 3.1 Study population and cohort split

**Figure 1:**
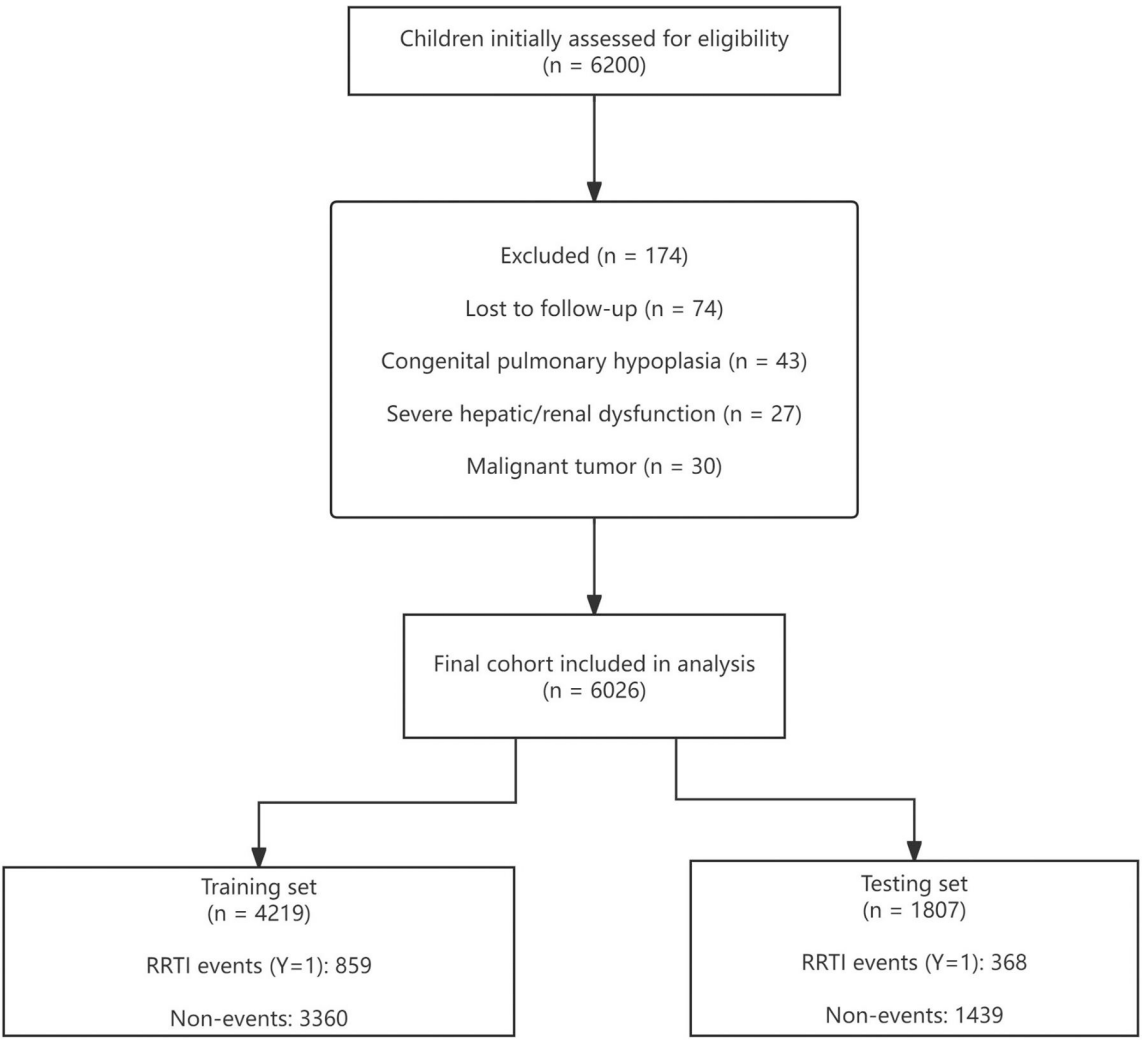
Study flowchart and cohort split. Flow diagram of participant screening and selection. Of the 6,200 children initially assessed for eligibility, 174 were excluded (lost to follow-up, *n* = 74; congenital pulmonary hypoplasia, *n* = 43; severe hepatic/renal dysfunction, *n* = 27; malignant tumor, *n* = 30), leaving 6,026 children in the final analytic cohort. The cohort was randomly divided into a training set (*n* = 4,219; RRTI events = 859; non-events = 3,360) and a testing set (*n* = 1,807; RRTI events = 368; non-events = 1,439).

A total of 6,200 children were initially assessed for eligibility. After 174 were excluded (lost to follow-up, *n* = 74; congenital pulmonary hypoplasia, *n* = 43; severe hepatic/renal dysfunction, *n* = 27; malignant tumor, *n* = 30), 6,026 children were included in the final analytic cohort (Figure 1). Overall, 1,227 children (20.4%) developed RRTIs during follow-up. The cohort was randomly divided into a training set (*n* = 4,219; events=859) and a testing set (*n* = 1,807; events = 368), with comparable event rates in the two sets.

### 3.2 Baseline clinical characteristics in the training set

Table 1 summarizes the baseline characteristics of children with and without RRTIs in the training set. Children with RRTIs had a lower birth weight (3.03 ± 0.53 vs. 3.42 ± 0.57 kg, *P* < 0.001) and a slightly lower BMI (15.84 ± 0.97 vs. 15.92 ± 0.94 kg/m^2^, *P* = 0.031). The RRTI group also had a higher rate of preterm birth (235 vs. 798, *P* = 0.032). In addition, children with RRTIs had slightly shorter breastfeeding duration and outdoor activity time (3.74 ± 0.67 vs. 3.80 ± 0.65 h/day, *P* = 0.029; 6.49 ± 0.88 vs. 6.56 ± 0.93 months, *P* = 0.035). Passive smoking exposure was significantly more common in the RRTI group (474 vs. 1,243, *P* < 0.001). Children with RRTIs were also more likely to have a history of allergy (409 vs. 526, *P* < 0.001) and asthma (117 vs. 167, *P* < 0.001). Urban-rural residence was also examined as an environmental factor during baseline comparison and candidate predictor assessment, but it was not retained in the final multivariable model. Furthermore, the distribution of infection sites differed significantly between the two groups (*P* < 0.001), with more upper respiratory and mixed/other presentations in the RRTI group (Table 1).

**Table 1:** Comparison of baseline clinical characteristics between recurrent RTI and non-recurrent respiratory tract infection in the training set.

Factor	Recurrent RTI (*n* = 859)	Non-recurrent RTI (*n* = 3,360)	*χ*²/t	*P* value
Sex (*n*)			0.705	0.401
Male	652	2,501		
Female	207	859		
Birth weight (kg)	3.03 ± 0.53	3.42 ± 0.57	−18.766	<0.001
BMI (kg/m²)	15.84 ± 0.97	15.92 ± 0.94	−2.154	0.031
Age (years)	6.39 ± 0.63	6.41 ± 0.64	−0.744	0.457
Family history of chronic respiratory disease (*n*)	167	746	2.915	0.088
Delivery mode (*n*)			2.318	0.128
Cesarean section	245	1,051		
Vaginal delivery	614	2,309		
Asphyxia at birth (*n*)			0.236	0.627
Yes	118	438		
No	741	2,922		
Preterm birth (*n*)	235	798	4.622	0.032
Outdoor activity time (h/day)	3.74 ± 0.67	3.80 ± 0.65	−2.191	0.029
Sleep time (h/day)	10.49 ± 2.55	10.49 ± 2.43	0.022	0.982
Breastfeeding duration (months)	6.49 ± 0.88	6.56 ± 0.93	−2.110	0.035
Passive smoking (*n*)	474	1,243	92.999	<0.001
Residence (*n*)			0.807	0.369
Rural	282	1,047		
Urban	577	2,313		
History of asthma (*n*)	117	167	80.162	<0.001
History of allergy (*n*)	409	526	403.198	<0.001
Infection site (*n*)			476.025	<0.001
Upper RTI	423	1,648		
Lower RTI	321	1,712		
Other/unknown	115	0		

### 3.3 Nutritional indicators associated with RRTIs

Comparisons of nutritional and hematologic indicators are shown in Table 2. Vitamin A and vitamin D levels were lower in the RRTI group (vitamin A: 0.35 ± 0.08 vs. 0.43 ± 0.11 mg/L, *P* < 0.001; vitamin D: 26.99 ± 9.07 vs. 32.32 ± 9.89 ng/mL, *P* < 0.001). Hemoglobin levels were also lower among children with RRTIs (115.20 ± 8.69 vs. 122.26 ± 9.53 g/L, *P* < 0.001). Other measured micronutrient indices, including zinc, iron, calcium, and magnesium, did not differ significantly between the groups (Table 2).

**Table 2:** Comparison of nutritional indicators between recurrent RTI and non-recurrent RTI in the training set (mean ± SD).

Indicator	Recurrent RTI (*n* = 859)	Non-recurrent RTI (*n* = 3,360)	*t*	*P* value
Serum zinc (*μ*mg/L)	76.26 ± 7.30	76.22 ± 7.53	0.120	0.905
Serum iron (μmg/L)	7.64 ± 0.86	7.69 ± 0.85	−1.306	0.192
Serum calcium (μmg/L)	1.63 ± 0.18	1.64 ± 0.19	−0.582	0.561
Serum magnesium (μmg/L)	1.59 ± 0.21	1.59 ± 0.22	−0.504	0.615
Vitamin A (mg/L)	0.35 ± 0.08	0.43 ± 0.11	−24.236	<0.001
Vitamin D (ng/mL)	26.99 ± 9.07	32.32 ± 9.89	−15.068	<0.001
Hemoglobin (g/L)	115.20 ± 8.69	122.26 ± 9.53	−20.851	<0.001

### 3.4 Independent predictors and nomogram development

**Figure 2:**
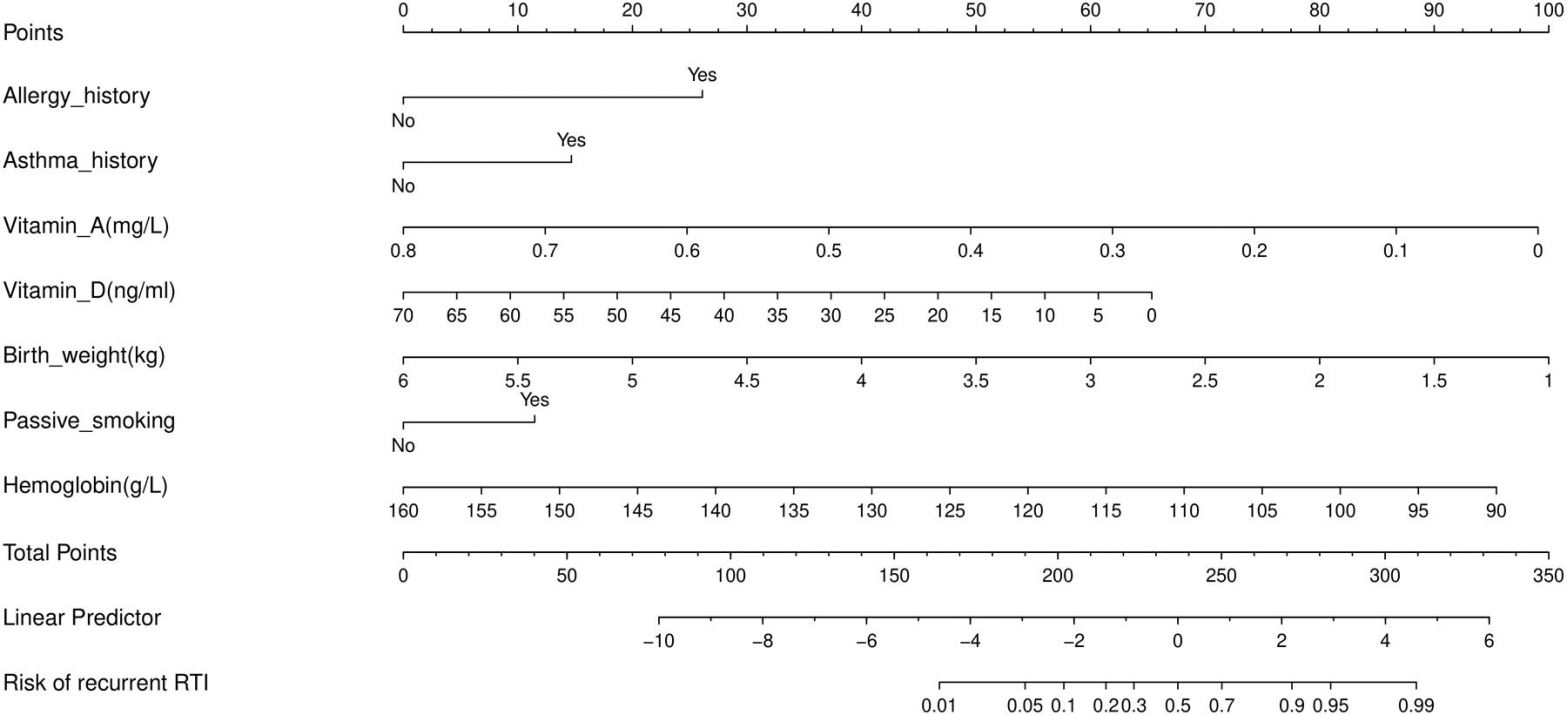
Nomogram for predicting 1-year risk of recurrent respiratory tract infections in children. A multivariable logistic regression-based nomogram incorporating seven independent predictors (history of allergy, history of asthma, vitamin A, vitamin D, birth weight, passive smoking exposure, and hemoglobin). For each predictor, draw a vertical line upward to the “Points” axis to assign points; sum the points to obtain “Total Points,” and then project downward to estimate the individualized probability of RRTI within 1 year.

Seven independent predictors of RRTIs were identified in the training set using multivariable logistic regression (Table 3). Although the eligibility criterion allowed inclusion of children aged 14 years or younger, the final analytic cohort was concentrated within a narrower observed age range (4.30–8.68 years; mean age, 6.41 ± 0.64 years). In an exploratory age-group analysis based on the observed age distribution (<5, 5 to <6, 6 to <7, and ≥7 years), the incidence of RRTIs was 25.6%, 20.4%, 20.2%, and 20.4%, respectively, with no significant between-group difference (*P* = 0.684). Consistently, age was not significantly associated with RRTIs when evaluated as a continuous predictor in univariable analysis (OR 0.983, 95% CI 0.891–1.084; *P* = 0.729). The strongest clinical predictors were a history of allergy (OR 5.187, 95% CI 4.226–6.366) and asthma (OR 2.522, 95% CI 1.821–3.493). Passive smoking exposure was also associated with an increased risk of RRTIs (OR 2.061, 95% CI 1.703–2.494). Protective factors included higher hemoglobin level (OR 0.425 per 10 g/L, 95% CI 0.381–0.474), higher vitamin D level (OR 0.556 per 10 ng/mL, 95% CI 0.500–0.611), and higher birth weight (OR 0.283 per kg, 95% CI 0.237–0.338). Vitamin A level was also inversely associated with RRTI risk (OR 0.458 per 0.1 mg/L increase, 95% CI 0.414–0.501). A nomogram was constructed on the basis of the final model (Figure 2).

**Table 3:** Multivariable logistic regression analysis for recurrent RTI in the training set.

Factor	B	SE	Wald χ²	*P* value	OR	95% CI
History of allergy	1.646	0.105	248.072	<0.001	5.187	4.226–6.366
History of asthma	0.925	0.166	31.020	<0.001	2.522	1.821–3.493
Vitamin A (per 0.1 mg/L)	−0.781	0.052	227.357	<0.001	0.458	0.414–0.501
Vitamin D (per 10 ng/mL)	−0.590	0.050	128.963	<0.001	0.556	0.500–0.611
Birth weight	−1.261	0.090	194.438	<0.001	0.283	0.237–0.338
Passive smoking	0.723	0.097	55.225	<0.001	2.061	1.703–2.494
Hemoglobin (per 10 g/L)	−0.860	0.060	239.942	<0.001	0.425	0.381–0.474

### 3.5 Model performance, calibration, clinical utility, and risk stratification

**Figure 3:**
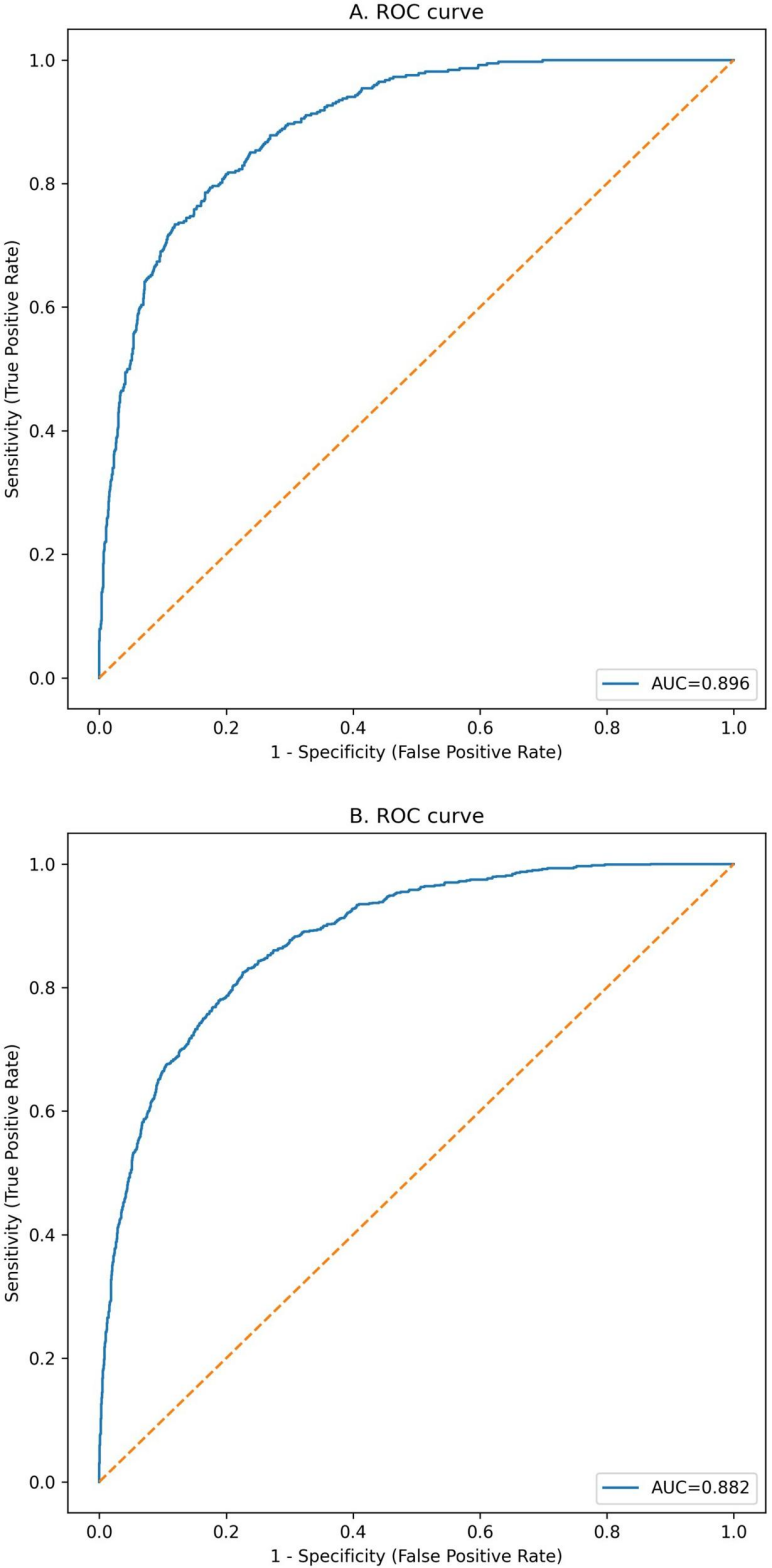
Receiver operating characteristic (ROC) curves of the nomogram in the testing and training sets. **(A)** ROC curve in the testing set (AUC = 0.896, 95% CI 0.879-0.912). **(B)** ROC curve in the training set (AUC = 0.882, 95% CI 0.870-0.894). AUC, area under the curve.

**Figure 4:**
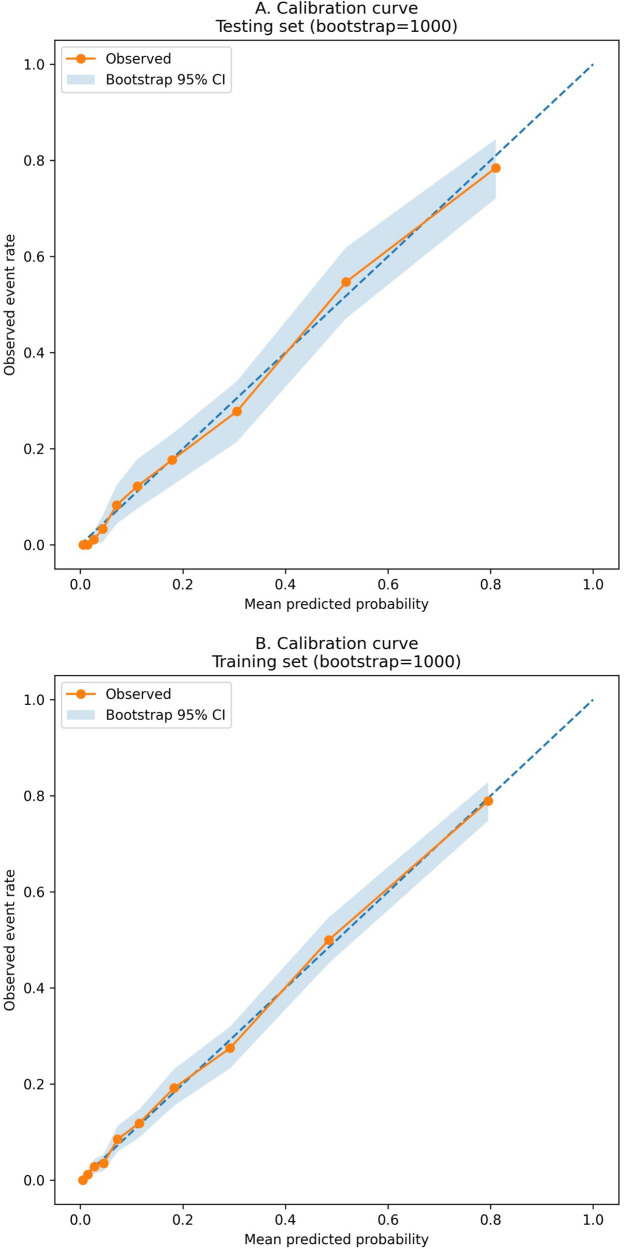
Calibration curves of the nomogram in the testing and training sets. Calibration plots comparing predicted probabilities with observed event rates. **(A)** Testing set. **(B)** Training set (bootstrap resampling for internal validation, 1,000 iterations). The diagonal line indicates perfect calibration, and deviation from the diagonal reflects miscalibration.

**Figure 5:**
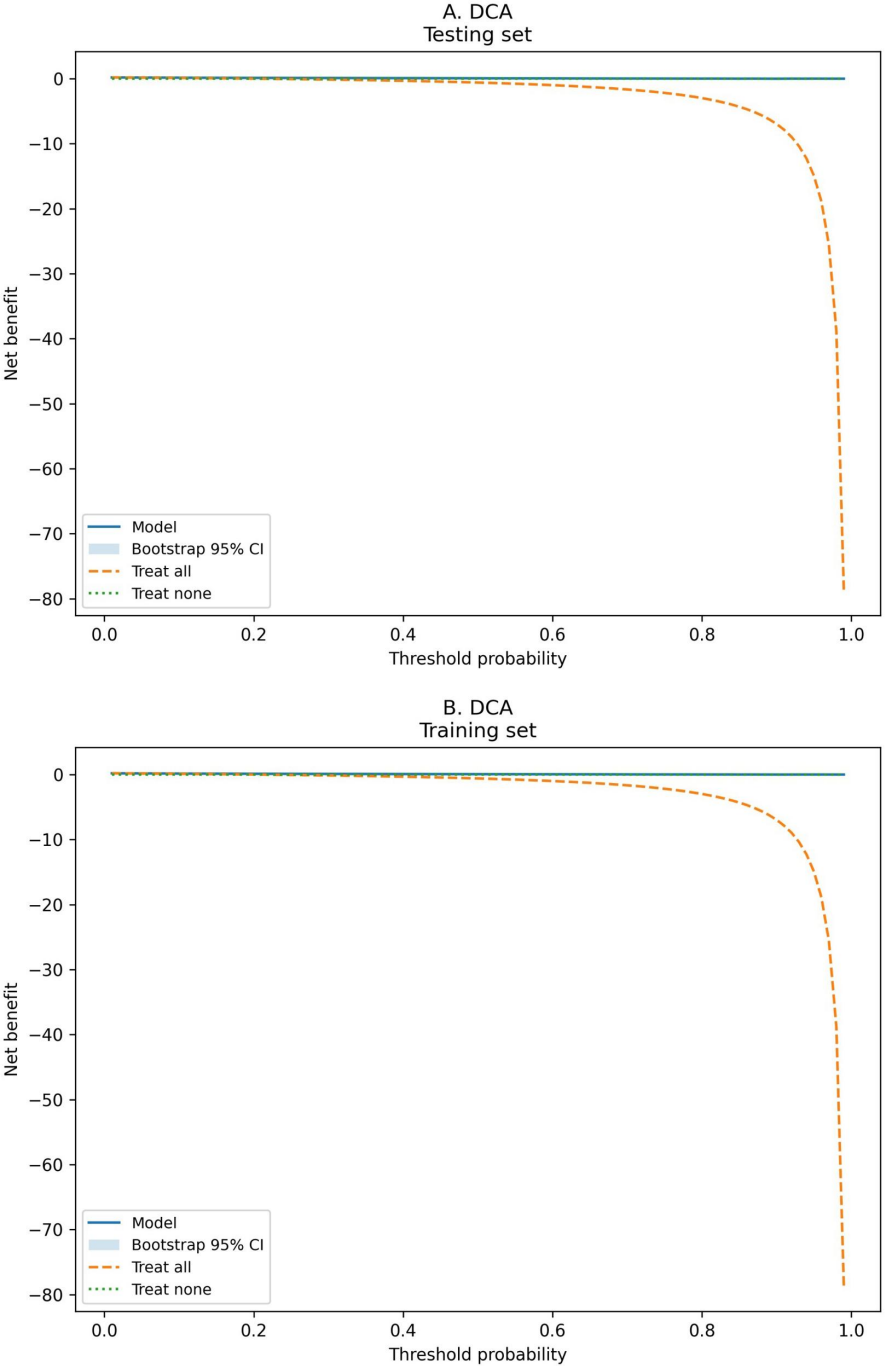
Decision curve analysis (DCA) of the nomogram in the testing and training sets. Decision-curve analysis evaluating clinical utility by quantifying net benefit across a range of threshold probabilities. **(A)** Testing set. **(B)** Training set. A higher net benefit indicates greater clinical usefulness compared with the default strategies of treating all or treating none.

**Figure 6:**
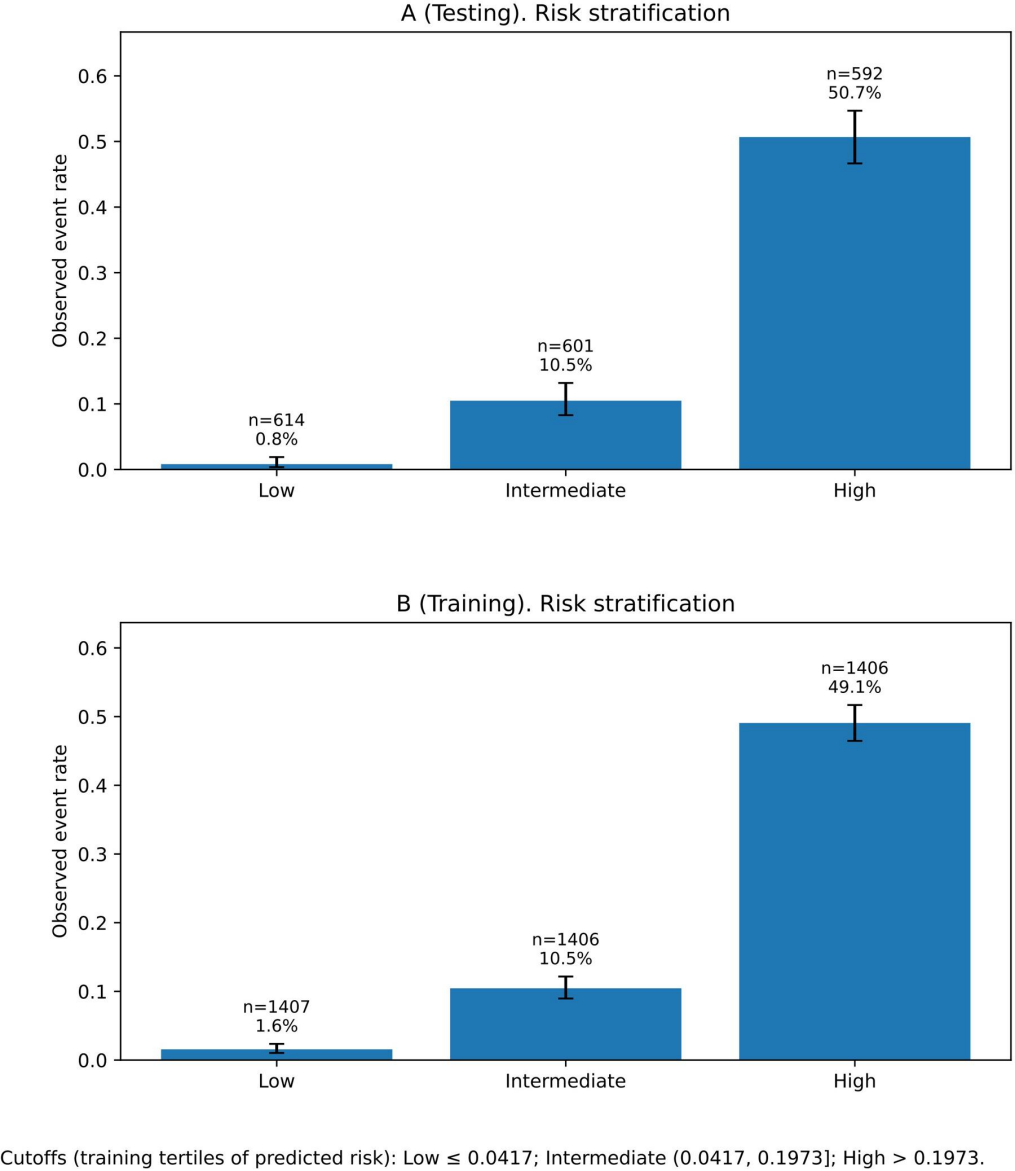
Risk stratification performance of the nomogram in the testing and training sets. Observed RRTI event rates across low-, intermediate-, and high-risk groups defined by predicted-probability cutoffs derived from training-set tertiles (low ≤ 0.0417; intermediate 0.0417-0.1973; high > 0.1973). **(A)** Testing set: event rates of 0.81% (low), 10.48% (intermediate), and 50.68% (high). **(B)** Training set: event rates of 1.56% (low), 10.46% (intermediate), and 49.08% (high).

The model showed good discrimination in both sets (Supplementary Table S1). The AUC was 0.882 (95% CI 0.870–0.894) in the training set and 0.896 (95% CI 0.879–0.912) in the testing set (Figure 3). Brier scores were 0.102 and 0.097, indicating good overall accuracy. Using the Youden index, sensitivity and specificity were 0.824 and 0.774 in the training set and 0.793 and 0.826 in the testing set, respectively (Supplementary Table S1). Quantile-based calibration data with bootstrap confidence intervals (Supplementary Table S2) were consistent with the calibration plots, which showed good agreement between predicted and observed risks in both the training and testing sets (Figure 4). Decision-curve analysis demonstrated favorable net benefit across clinically relevant threshold probabilities, with bootstrap confidence intervals reported for selected thresholds (e.g., threshold 0.10 in the testing set: net benefit 0.157, 95% CI 0.137–0.176; Supplementary Table S3) (Figure 5). Predicted probabilities were further stratified into low-, intermediate-, and high-risk groups using tertiles derived from the training set for clinical interpretability (low ≤ 0.041666; intermediate 0.041666–0.197340; high > 0.197340). In the testing set, the observed event rates were 0.81% in the low-risk group, 10.48% in the intermediate-risk group, and 50.68% in the high-risk group, indicating clear separation among the risk strata (Table 4; Figure 6). Comparable gradients were observed in the training set (1.56%, 10.46%, and 49.08%, respectively; Table 4).

**Table 4:** Risk stratification by predicted probability (cutoffs from training tertiles).

Set	Risk group	N	Events (Y = 1)	Observed event rate	95% CI (Wilson)	Mean predicted risk	Predicted risk range
Testing (Panel A)	Low	614	5	0.0081	0.0035–0.0189	0.0178	0.0007–0.0416
Testing (Panel A)	Intermediate	601	63	0.1048	0.0828–0.1319	0.0993	0.0417–0.1961
Testing (Panel A)	High	592	300	0.5068	0.4666–0.5469	0.5172	0.1978–0.9866
Training (Panel B)	Low	1,407	22	0.0156	0.0103–0.0236	0.0181	0.0002–0.0417
Training (Panel B)	Intermediate	1,406	147	0.1046	0.0896–0.1216	0.0999	0.0417–0.1973
Training (Panel B)	High	1,406	690	0.4908	0.4647–0.5169	0.4929	0.1979–0.9958

Cutoffs (derived from training set tertiles of predicted risk): Low ≤ 0.041666; Intermediate (0.041666, 0.197340); High > 0.197340.

## 4 Discussion

### 4.1 Principal findings

In this decade-long real-world cohort of children with respiratory tract infections, we developed and validated a customized risk-stratification nomogram for predicting the 1-year risk of recurrent respiratory tract infections (RRTIs). The final model included seven independent predictors that are readily available in routine clinical practice: history of allergy, history of asthma, passive smoking exposure, vitamin A level, vitamin D level, hemoglobin level, and birth weight. The nomogram showed strong discrimination and good calibration in both the training and testing sets, and decision-curve analysis demonstrated a favorable net benefit across clinically relevant threshold probabilities. Importantly, the proposed risk-stratification approach separated children into low-, intermediate-, and high-risk groups with markedly different observed event rates, supporting its potential value in frontline pediatric decision-making.

### 4.2 Interpretation and comparison with prior evidence

Our findings are consistent with the multifactorial pathophysiology of RRTIs and are biologically plausible. First, allergy and asthma were the strongest clinical predictors (4, 17, 18), which is consistent with the concept that chronic airway inflammation and epithelial barrier dysfunction may impair mucosal defense, facilitate pathogen adherence to mucosal surfaces, and increase susceptibility to recurrent infections. In clinical practice, children with atopy or asthma often exhibit increased airway reactivity, mucus hypersecretion, and altered local immune responses, all of which may contribute to recurrent respiratory symptoms and infection-like episodes. Second, passive smoking exposure remained independently associated with RRTIs. Secondhand smoke can damage airway cilia (19–21), increase airway inflammation, and impair innate and adaptive immune responses, thereby increasing susceptibility to respiratory pathogens.

Nutritional and hematologic indicators also played an important role. Low vitamin A and vitamin D levels were both associated with a higher risk of RRTIs. Vitamin A is essential for epithelial integrity and immune regulation, and deficiency may disrupt mucosal barriers and alter neutrophil and macrophage function. Vitamin D has immunomodulatory effects (7, 22–24), including the induction of antimicrobial peptides and the regulation of inflammatory responses, which may help limit pathogen replication and tissue injury. Lower hemoglobin level was another independent predictor and may reflect underlying iron deficiency, overall nutritional vulnerability, or chronic inflammatory burden. Finally, lower birth weight was associated with increased risk, possibly reflecting early-life developmental disadvantage and reduced immune reserve. Taken together, these predictors support a coherent risk profile integrating atopic predisposition, environmental exposure, and nutritional and physiologic resilience.

### 4.3 Clinical implications

The nomogram may facilitate practical, risk-adapted prevention strategies. For children identified as high risk, clinicians may consider (1) reinforced caregiver education and reduction of household smoke exposure; (2) closer follow-up for children with atopy or asthma, with optimization of controller therapy and guidance on trigger avoidance; and (3) targeted assessment and correction of nutritional and hematologic deficits, such as evaluating vitamin A/vitamin D status and anemia, followed by individualized supplementation or dietary interventions when clinically indicated. For children at intermediate risk, the model may support shared decision-making regarding monitoring intensity and preventive counseling. Notably, this risk-stratification framework may help prioritize resources, particularly in high-volume pediatric settings (25).

### 4.4 Strengths

This study has several strengths. It was based on a large real-world cohort with a 10-year inclusion window and a clearly defined 1-year follow-up period. The predictors were readily available and clinically interpretable, thereby enhancing both feasibility of implementation and potential generalizability. In addition to discrimination, we evaluated calibration and clinical utility using decision-curve analysis. Furthermore, we presented an explicit risk-stratification system, which is consistent with the translational emphasis and clinical applicability valued in Frontiers publications (26).

### 4.5 Limitations and future directions

Several limitations should be acknowledged. First, this was a single-center observational cohort, and the model may require recalibration before use in populations with different case mixes, healthcare access, or nutritional patterns. Second, although hold-out validation was performed, further external validation across multiple centers and regions is needed to confirm transportability. Third, the outcome definition relied on age-specific episode counts during follow-up; therefore, misclassification is possible if infections were managed outside our system or if reporting was incomplete, although standardized follow-up procedures were applied. Fourth, some potentially relevant predictors, such as daycare attendance, vaccination status, indoor air pollution, antibiotic exposure, and detailed immunologic testing, were not included. Because congenital immunodeficiency was not recorded as a fully standardized variable across the entire cohort, we were unable to reliably quantify how many children had documented congenital immunodeficiency retrospectively; therefore, residual confounding from unrecognized or incompletely documented immune disorders cannot be entirely excluded. Although residence (urban vs. rural) was collected and examined as an environmental factor, our dataset did not capture more granular environmental context, such as household crowding, ambient air quality, or neighborhood-level socioeconomic conditions. Fifth, although the prespecified eligibility criterion was ≤14 years, the actual cohort was concentrated between approximately 4 and 9 years of age; therefore, the applicability of the model to infants, toddlers, and older adolescents should be interpreted cautiously and requires further validation in broader pediatric populations. Future studies should examine whether adding these variables can meaningfully improve performance without compromising feasibility. In addition, several index-episode management/severity indicators that may reflect short-term illness burden-such as hospitalization status, chest x-ray assessment, oxygen requirement, length of stay, and antibiotic use-were not available in a complete and standardized manner and therefore could not be analyzed. Future work should focus on multicenter validation, assessment of temporal stability, and implementation research. Integrating the nomogram into a web-based calculator or electronic medical record tool and testing whether risk-guided interventions reduce the burden of RRTIs would be important next steps. In addition, exploring nonlinear effects and interactions, such as those between vitamin status and atopy or smoke exposure, may further refine individualized risk estimation. In addition, because microbiological etiologic testing was not routinely standardized across all children in this retrospective cohort, we were unable to present reliable proportions of viral and bacterial respiratory tract infections.

## 5 Conclusion

We developed and validated a customized nomogram for predicting the 1-year risk of recurrent respiratory tract infections in a large, decade-long real-world pediatric cohort. By integrating readily available clinical history (allergy and asthma), environmental exposure (passive smoking exposure), and nutritional/physiologic indicators (vitamin A, vitamin D, hemoglobin, and birth weight), the model demonstrated good discrimination, calibration, and clinical utility. This tool may support early risk stratification and targeted preventive strategies in routine pediatric practice.

## Data Availability

The raw data supporting the conclusions of this article will be made available by the authors, without undue reservation.
