# Supplementary material for: A decade-long real-world cohort (2016–2025): development of an individualized risk-stratification nomogram and evaluation of clinical utility for recurrent respiratory tract infections in children
Source: Front Pediatr. 2026 May 12;14:1806366. doi: 10.3389/fped.2026.1806366 (PMC13201507; doi:10.3389/fped.2026.1806366)
Supplement: Supplementary file 1 [file Table1.docx]

**Supplementary Table S1. Performance metrics (ROC/AUC and Brier score)**

| Set | N | Events (Y=1) | Event rate | AUC | AUC 95% CI (bootstrap=1000) | Youden threshold | Sensitivity | Specificity | Brier score |
| --- | --- | --- | --- | --- | --- | --- | --- | --- | --- |
| Testing (Panel A) | 1807 | 368 | 0.204 | 0.896 | 0.879–0.912 | 0.231 | 0.793 | 0.826 | 0.097 |
| Training (Panel B) | 4219 | 859 | 0.204 | 0.882 | 0.870–0.894 | 0.184 | 0.824 | 0.774 | 0.102 |
