# Supplementary material for: A decade-long real-world cohort (2016–2025): development of an individualized risk-stratification nomogram and evaluation of clinical utility for recurrent respiratory tract infections in children
Source: Front Pediatr. 2026 May 12;14:1806366. doi: 10.3389/fped.2026.1806366 (PMC13201507; doi:10.3389/fped.2026.1806366)
Supplement: Supplementary file 2 [file Table2.docx]

**Supplementary Table S2. Calibration curve data (10 quantile bins; observed vs predicted with 95% bootstrap CI)**

| Set | Bin | Mean predicted | Observed event rate | 95% CI (bootstrap=1000) |
| --- | --- | --- | --- | --- |
| Testing (Panel A) | 1 | 0.005 | 0.000 | 0.000–0.000 |
| Testing (Panel A) | 2 | 0.014 | 0.000 | 0.000–0.000 |
| Testing (Panel A) | 3 | 0.026 | 0.011 | 0.000–0.030 |
| Testing (Panel A) | 4 | 0.044 | 0.033 | 0.011–0.060 |
| Testing (Panel A) | 5 | 0.071 | 0.083 | 0.043–0.126 |
| Testing (Panel A) | 6 | 0.112 | 0.122 | 0.077–0.171 |
| Testing (Panel A) | 7 | 0.179 | 0.177 | 0.125–0.234 |
| Testing (Panel A) | 8 | 0.305 | 0.278 | 0.215–0.342 |
| Testing (Panel A) | 9 | 0.518 | 0.547 | 0.476–0.619 |
| Testing (Panel A) | 10 | 0.810 | 0.785 | 0.725–0.843 |
| Training (Panel B) | 1 | 0.005 | 0.000 | 0.000–0.000 |
| Training (Panel B) | 2 | 0.015 | 0.012 | 0.002–0.023 |
| Training (Panel B) | 3 | 0.028 | 0.028 | 0.014–0.045 |
| Training (Panel B) | 4 | 0.046 | 0.036 | 0.019–0.053 |
| Training (Panel B) | 5 | 0.073 | 0.085 | 0.062–0.112 |
| Training (Panel B) | 6 | 0.115 | 0.119 | 0.089–0.150 |
| Training (Panel B) | 7 | 0.183 | 0.192 | 0.155–0.231 |
| Training (Panel B) | 8 | 0.292 | 0.275 | 0.235–0.321 |
| Training (Panel B) | 9 | 0.485 | 0.500 | 0.454–0.548 |
| Training (Panel B) | 10 | 0.795 | 0.789 | 0.747–0.825 |
