# Supplementary material for: A decade-long real-world cohort (2016–2025): development of an individualized risk-stratification nomogram and evaluation of clinical utility for recurrent respiratory tract infections in children
Source: Front Pediatr. 2026 May 12;14:1806366. doi: 10.3389/fped.2026.1806366 (PMC13201507; doi:10.3389/fped.2026.1806366)
Supplement: Supplementary file 3 [file Table3.docx]

**Supplementary Table S3. DCA net benefit at selected thresholds (with 95% bootstrap CI; B=300)**

| Threshold | Testing: Model NB | Testing: 95% CI | Testing: Treat-all NB | Training: Model NB | Training: 95% CI | Training: Treat-all NB |
| --- | --- | --- | --- | --- | --- | --- |
| 0.05 | 0.178 | 0.158–0.196 | 0.162 | 0.174 | 0.162–0.185 | 0.162 |
| 0.10 | 0.157 | 0.137–0.176 | 0.115 | 0.151 | 0.139–0.163 | 0.115 |
| 0.20 | 0.126 | 0.106–0.144 | 0.005 | 0.120 | 0.108–0.132 | 0.005 |
| 0.30 | 0.107 | 0.087–0.126 | -0.138 | 0.099 | 0.087–0.109 | -0.138 |
| 0.50 | 0.070 | 0.050–0.088 | -0.593 | 0.062 | 0.051–0.073 | -0.593 |
| 0.70 | 0.029 | 0.008–0.049 | -1.654 | 0.028 | 0.016–0.038 | -1.655 |
| 0.90 | -0.005 | -0.030–0.015 | -6.963 | 0.002 | -0.012–0.012 | -6.964 |
| 0.95 | 0.011 | 0.006–0.015 | -14.927 | 0.004 | -0.007–0.011 | -14.928 |
